# The marketing of “stem cell” supplements on Amazon.com: Assessing alignment with regulatory frameworks in the United States and Canada

**DOI:** 10.1016/j.stemcr.2025.102675

**Published:** 2025-10-09

**Authors:** Alessandro R. Marcon, Marco Zenone, Vincenza Boniface, Sophie Sigfstead, Blake Murdoch, Timothy Caulfield

**Affiliations:** 1 Health Law Institute, Office 470, Faculty of Law, University of Alberta, Edmonton, AB T6G 2H5, Canada; 2 Faculty of Health Sciences, University of Ottawa, Ottawa, ON K1H 8M5, Canada

## Abstract

The direct-to-consumer marketing of stem cell interventions now includes an emerging ecommerce marketplace of “stem cell” supplements. This research assessed the marketing of stem cell supplements on Amazon.com, evaluating how that marketing aligned or conflicted with regulatory frameworks in the United States and Canada. Given the results, new regulation strategies should be considered to offer greater transparency and consumer protection.

## Introduction

Scientifically unsupported commercial activity has long accompanied developments in stem cell research. Clinics’ online marketing of stem cell treatments for illness, pain, repair, and injury has been studied extensively worldwide, consistently reporting unproven and inflated health benefit claims (Lyons et al., 2021; Turner et al., 2024). Discourse in, but not limited to, these commercial spaces often distorts, exaggerates, and manipulates scientific evidence and rhetoric to sell stem-cell products, therapies, and ideas (Turner et al., 2024; Ellythy et al., 2025)—a phenomenon called science hype or “scienceploitation” (Caulfield et al., 2019). Misrepresenting scientific legitimacy breaches ethical guidelines and exploits consumers. In response, calls exist for increased regulation, including from the International Society for Stem Cell Research (ISSCR) calling for “accurate, current, balanced, and responsive public representations of stem cell research” (ISSCR, 2021). Effective regulation has been challenging, however, as online cross-border marketing can evolve quickly, challenge localized regulatory frameworks, and present with opaque platform mechanisms (Lyons et al., 2021; Turner et al., 2024), such as the obfuscation of paid-promotion content and non-transparent black box algorithms governing platform activity.

The marketplace of stem cell therapies and products continues to evolve. Recently, “stem cell” supplements have emerged in online marketplaces. Supplements remain popular in North America and abroad (Knoepfler, 2024; Sullivan, 2025). Consisting typically of pills, powders, or liquids, supplements are promoted to augment one’s diet with nutrients such as herbs, probiotics, amino acids, enzymes, vitamins, or minerals. They form a core component of the highly profitable wellness industry, where individual health is promoted as an individual responsibility to achieve ever-shifting ideas of optimization (Derkatch, 2022). In these contexts, wellness advocates promoting supplements commonly view regulatory authorities as restricting autonomous health promotion and contributing to society’s toxicity, in large part by enabling the pharmaceutical industry (Derkatch, 2022). Despite sharing industry connections, supplements, in contrast to pharmaceuticals, are commonly viewed among wellness advocates as natural, pure, and safe (Derkatch, 2022). Supplements, however, may suffer from labeling inaccuracies, offer few health benefits, save nutrient deficiency cases, and lead to health risks (Binns et al., 2018).

Consumer desire for, and profitability of, supplements creates complex challenges for regulatory bodies (Sullivan, 2025; Derkatch, 2022; Jarry, 2023). In Canada and the United States, where supplements are classified as Natural Health Products (NHPs) and dietary supplements, respectively, regulatory efforts have been criticized for ineffectiveness while also being met with resistance from industry and the public (Sullivan, 2025; Derkatch, 2022; Jarry, 2023). Currently, dietary supplements are not subjected to regulatory approval—in terms of safety or efficacy—before gaining market access in the United States. In contrast, market entry for NHPs in Canada requires product and manufacturing site licenses from Health Canada (see supplemental information [SM 1] for detailed comparative analysis of both frameworks) (Health Canada, 2012; U.S. Food and Drug Administration, 2024; US FTC, 2024). Aside from this key difference, both overarching regulatory parameters are similar (Jarry, 2023). In both jurisdictions, industry is prohibited from making strong causal claims around a products’ ability to diagnose, prevent, cure, or mitigate serious ailments and disease (Health Canada, 2012; U.S. Food and Drug Administration, 2024; US FTC, 2024). In the United States, all dietary supplements marketed with a health claim are required to include the following disclaimer: “This statement has not been evaluated by the Food and Drug Administration. This product is not intended to diagnose, treat, cure, or prevent any disease” (U.S. Food and Drug Administration, 2024).

Similar regulatory measures in both jurisdictions detail how a product’s health claims need substantiation with sufficient evidence and must not be marketed by distorting the scientific evidence base (Health Canada, 2012; U.S. Food and Drug Administration, 2024; US FTC, 2024). Products that claim greater efficacy, specifically in more serious health contexts, would—as described—require a stronger evidentiary base. However, while both jurisdictions present consumer-protecting regulatory measures against misleading claims, both permit industry to market products’ health claims using vague, indirect, or hedged language, via “structure/function” claims in the United States and “general health claims” in Canada (SM 1). Both claim types broadly apply to lower risk health applications and permit a product’s mechanism of action to be described with non-direct verbs (e.g., “promote, “maintain,” “support,” and “help”) or with causality expressed with modality (e.g., “may,” “could,” and “might”). Product marketing is thus permissible if it is only suggestive of potential benefit and if the relevant substantiating scientific evidence-base consensus is not presented misleadingly (SM 1).

Supplement regulatory breaches have been observed in both Canada and the United States. In the US, the Federal Trade Commission (FTC), which coordinates with the Food and Drug Administration (FDA) but addresses advertising discourse, has settled or adjudicated over 200 cases “involving false or misleading advertising claims about the benefits or safety of dietary supplements or other health-related products” (US FTC, 2024). In Canada, the Office of the Auditor General’s audit of Health Canada’s NHP regulation program found that “Canada fell short of ensuring that products were safe and effective” (Office of the Auditor General of Canada, 2021). Issues were found in both approval and monitoring processes, noting that “little” was done “to prevent poor information from being given to consumers about licensed natural health products” (Office of the Auditor General of Canada, 2021). Additionally, analysis on a sample of licensed NHPs found misleading information in 88% of products’ labels and 56% of product’s health claims and consumer uses (Office of the Auditor General of Canada, 2021).

Funded to investigate emerging trends in regenerative medicine commercialization, we assessed these regulatory frameworks in the context of emerging stem cell supplements. As the placement and storage of human stem cells in pills, liquids, or capsules is scientifically implausible, we sought to investigate how stem cell supplements were defined and promoted in popular online marketplaces. In December 2023, we built a dataset of all stem cell products found on the ecommerce platform Amazon.com in the category “Vitamins, Minerals, and Supplements.” Our finalized dataset of 184 stem cell supplement listings from 133 companies included only unique (non-duplicated) products with a described stem cell-related function. Coding captured health ailments addressed by products and corresponding rhetoric in health claims, use of beneficial product descriptors, and the presence of scientific rhetoric as well as health care practitioners/scientists (see SM 2 for a complete methods overview). Capturing the presence of scientific rhetoric/evidence and health care practitioners (i.e., appeals to scientific expertise) was included as a metric for assessing science hype or scienceploitation (Caulfield et al., 2019). Stratified descriptive statistical analysis was performed between products available in Canada and the United States versus only in the United States.

## How stem cell supplements were marketed on Amazon.com

The stem cell supplement advertisements on Amazon.com (*n* = 184) (Table S1) claimed that products either contained stem cells, created more stem cells, or improved stem cell functioning. These ads sometimes defined stem cells or described their functionality (61, 33.2%) but almost always made health claims in relation to particular health ailments (173, 94.0%). Listings commonly made claims with regard to multiple health ailments/contexts (Table 1). Beneficial product descriptors were listed in the majority of products (155, 84.2%), and over 40% of all products made an explicit mention of science or scientific evidence in relation to product quality or efficacy (e.g., “cutting-edge science,” “clinically tested,” or “scientifically proven”) (77, 41.8%). Health care professionals had a smaller but not negligible presence (65, 35.3%) (Table 1).

**Table 1:** “Stem cell” supplement information contained in Amazon.com listings (*N* = 184)

Information in product listing	Products available in US and Canada	Products available only in US	All products
Listed products^a^	**89 (48.4%)**	**95 (51.6%)**	**184 (100%)**
Defining characteristics of stem cells^b^	32 (36.0%)	29 (30.5%)	61 (33.2%)
Benefits listed for particular health ailments	84 (94.4%)	89 (93.7%)	173 (94.0%)
Mean average of health ailments per listing	4.6	5.1	4.8
Ads with ≥10 ailments	4 (4.5%)	9 (9.5%)	13 (7.1%)
≥5 ailments	45 (50.6%)	51 (53.6%)	96 (52.2%)
≥3 ailments	68 (76.4%)	73 (76.8%)	141 (76.6%)
Only 1 ailment	6 (6.7%)	10 (10.5%)	16 (8.7%)
Total number of health ailments	50	52	64
Health ailments listed by ranking (min 7% of total)			
Aging (anti-aging/healthy)	56 (62.9%)	47 (49.5%)	103 (56.0%)
Immunity	30 (33.7%)	47 (49.5%)	77 (41.8%)
Energy	36 (40.4%)	31 (32.6%)	67 (36.4%)
Healing/repair	31 (34.8%)	30 (31.6%)	61 (33.2%)
Skin	26 (29.2%)	26 (27.4%)	52 (28.3%)
Brain-related (e.g., cognitive functions, “brain fog,” “thinking,” and “focus”)	26 (29.2%)	20 (21.1%)	46 (25.0%)
Overall/general health	20 (22.5%)	25 (26.3%)	45 (24.5%)
Cardiovascular related	18 (20.2%)	23 (24.2%)	41 (22.3%)
Joints	12 (13.5%)	21 (22.5%)	33 (17.9%)
Antioxidant related	15 (16.9%)	16 (16.8%)	31 (16.8%)
Bones	6 (6.7%)	18 (18.9%)	24 (13.0%)
Muscles	11 (12.4%)	11 (11.6%)	22 (12.0%)
Inflammation	9 (10.1%)	12 (12.6%)	21 (11.4%)
Metabolism	12 (13.5%)	9 (9.5%)	21 (11.4%)
Hair	8 (9.0%)	10 (10.5%)	18 (9.8%)
Heart specific	8 (9.0%)	9 (9.5%)	17 (9.2%)
Memory specific	8 (9.0%)	8 (8.4%)	16 (8.7%)
Mood	4 (4.5%)	11 (11.6%)	15 (8.2%)
Stress	8 (9.0%)	5 (5.3%)	13 (7.1%)
Detailing of scientific support for quality/efficacy	**41 (46.1%)**	**36 (37.9%)**	**77 (41.8%)**
Inclusion of health care/health science professionals	**33 (37.1%)**	**32 (33.7%)**	**65 (35.3%)**
Beneficial product descriptors (min 6% of total)	**71 (79.8%)**	**84 (88.5%)**	**155 (84.2%)**
Natural	42 (47.2%)	61 (64.2%)	103 (56.0%)
GMO-free	32 (36.0%)	43 (45.3%)	75 (40.8%)
Gluten-free	31 (34.8%)	30 (31.6%)	61 (33.2%)
Pure/organic	28 (31.5%)	24 (25.3%)	52 (28.3%)
Vegan/vegetarian	21 (23.6%)	27 (28.4%)	48 (26.1%)
No fillers/preservatives	24 (27.0%)	21 (22.7%)	45 (24.5%)
Soy-free	12 (13.5%)	10 (10.4%)	22 (12.0%)
Ancestral/traditional product	5 (5.6%)	14 (14.7%)	19 (10.3%)
Toxin-free	4 (4.5%)	8 (8.4%)	12 (6.5%)

a Products listed as not currently available, not discontinued by the manufacturer, were included in the dataset as unavailability could relate to a lack of current supply in the process of being restocked.

b Products defining stem cells and/or describing their function in human anatomy.

Marketing portrayed beneficial effect for over 60 diverse ailments, most frequently regarding aging, immunity, energy, healing/repair, skin health, overall/general health, brain health, and cardiovascular functioning (Table 1). All health claims were expressed with vague, non-causal verbs such as “supports,” “promotes,” “bolsters,” “protects,” etc., alongside the use of asterisk-marked hedging statements, and modal verbs such as “may,” “could,” or “can.” The required FDA dietary supplement disclaimer appeared in all dietary supplement ads with a health claim. For beneficial product descriptors (155, 84.2%), products on average listed 2.5 characteristics indicative of wellness trends, the most used being “natural” (103, 56.0%), GMO-free (75, 40.8%), gluten-free (61, 33.2%), and pure/organic (52, 28.3%) (Table 1). No considerable trends were observed between products with differing availability between Canada and the United States (Table 1).

## Regulatory challenges and strengthening strategies

The marketing of stem cell supplements on Amazon.com highlights regulatory limitations and challenges. This stem cell supplement marketing did not explicitly mention serious, life-threatening diseases (e.g., cancer, asthma, arthritis, dementia, congestive heart failure, etc.) or use direct causal language of effect (e.g., “mitigate,” “prevent,” “cure,” or “treat”). Products were thus arguably aligned with the “general health claims” and “structure/function claims” in the respective Canadian and American regulatory frameworks. The current frameworks therefore permit and arguably enable free rein for vague (and scientifically unsupported) claims for general health ailments and also more serious ailments presented with discreet language.

There is a problematic and potentially dangerous assumption that consumers accurately interpret this marketing as only claiming minor therapeutic potential for non-serious conditions. For example, although “dementia” was absent in the marketing, anti-aging, memory, and cognitive function ailments were frequently mentioned. Explicit mentioning of disease prevention was absent, but text with “strengthening,” “supporting,” or “promoting” immunity was common. Further, this marketing arguably generates consumer impressions of ample scientific evidentiary support, at odds with the current state of stem cell therapeutics (Knoepfler, 2024). As detailed in the FTC’s 2022 “Health Products Compliance Guidance” document, which does not carry legal force or effect but offers explanations and interpretations of FTC advertising law, there is a regulatory need to interpret marketing discourse through reasonable consumer interpretations of an entire ad’s impression (US FTC, 2024). It stresses that it is prohibited for ads to give a misleading impression of scientific consensus (US FTC, 2024). There were no clear marketing differences between products available in Canada versus the United States. This is unsurprising, given the similarity of regulatory platforms aside from Canada’s unique licensing mandates. There are numerous reasons a company may not pursue Canadian markets, and future research could assess the potential impact of Canada’s licensing requirements.

Whether aligning with current regulations permitting vague marketing or conflicting with regulation prohibiting a distortion of the evidentiary base, stem cell supplements with no clear consumer benefit are being promoted and sold on Amazon.com. The question stands whether—and which kinds of—regulatory reform is needed in response. Supplement regulation in North America has been (ardently) resisted in the past (Derkatch, 2022; Binns et al., 2018; Jarry, 2023), and efforts to frame supplements as distinct from the perceived harms and dangers of “mainstream” pharmaceuticals (Derkatch, 2022) were observed in the marketing of stem cell supplements on Amazon.com (82% included common wellness characteristics, such as “naturalness” [56%]). Given historical efforts to restrict supplement access, there is good reason to believe that increasing regulation dramatically could face backlash from consumers and industry.

Regulatory frameworks, however, should not enable manufacturers to make inaccurate, unsubstantiated product claims. Health Canada has acknowledged its NHP regulatory limitations and is implementing changes to strengthen capacity (Office of the Auditor General of Canada, 2021). Notably, changes will increase licensing costs, helping reduce the financial burden on Health Canada, which may deter frivolous and egregiously inadequate applications. Tightening market access may lead to fewer examples of highly problematic marketing and facilitate post-market surveillance, including advertising with health claims and appeals to scientific efficacy. It would be a dramatic policy shift to regulate market-entry access (e.g., with licensing requirements) in the United States, yet doing so could enhance consumer safeguards.

Monitoring products’ marketing is labor- and time intensive. While a few hundred cases of supplement regulation have occurred in the United States, more than 100,000 dietary supplements are estimated to be available for consumers (Sullivan, 2025). The stem cell products observed in this study may not be classified as high risk, thus falling outside the urgency scope of regulators. And yet, a failure to regulate in any meaningful way hurts consumers by allowing the misleading marketing of deceptive products. It hinders understandings of, and trust in, stem cell science and related efforts for evidence-based policy making. Research shows that increased regulation of stem cell commercialization can offer increased consumer protections (Ikonomou et al., 2024). Approaches and mechanisms to surveil online marketing could be created with input from existing guidelines (ISSCR, 2021) that identify keywords associated with serious health contexts or vulnerable populations (e.g., the elderly or pregnant people). Uses of scientific rhetoric claiming evidentiary support—examples of which are described in this research—could trigger increased investigation, including requirements to produce corresponding evidence. Though complex, governments could begin working with automated AI systems to flag marketing cases for further investigation. Regulatory bodies must be sufficiently funded and mandated to regulate on the basis of scientific accuracies. Given the expanding market for supplements and wellness products—and the growing evidence of deceptive practices and potential harms—the creation of new regulatory strategies seems essential to strengthen consumers’ ability to make evidence-informed decisions.

## Acknowledgments

This research was funded by the 10.13039/501100000098Stem Cell Network (grant #917390) under the project title: Law, Public Policy and Social License for Next-Generation Regenerative Medicine. This funding agency played no role in any aspect of this research including the decision for publication. No authors were precluded from accessing data in the study, and all authors accept responsibility to submit the manuscript for publication. Authors wish to thank Robyn Hyde-Lay for managerial assistance with this research.

## Author contributions

A.R.M., M.Z., and T.C. designed the study. M.Z., A.R.M., and V.B. collected, organized, and managed data. A.R.M., M.Z., V.B., and S.S. analyzed and interpreted the data with input from B.M. and T.C. A.R.M. wrote the first draft of the manuscript. M.Z., V.B., S.S., B.M., and T.C. reviewed and edited the manuscript. All authors had access to the data throughout the research. A.R.M., M.Z., V.B., and S.S. verified the data reported in the manuscript.

## Declaration of interests

The authors declare no competing interests.

## Supplemental information

Document S1. Methods S1 and S2

Table S1. Final stem cell supplement Amazon data + coding

Document S2. Article plus supplemental information
