# Supplementary material for: The marketing of “stem cell” supplements on Amazon.com: Assessing alignment with regulatory frameworks in the United States and Canada
Source: Stem Cell Reports. 2025 Oct 9;20(11):102675. doi: 10.1016/j.stemcr.2025.102675 (PMC12790744; doi:10.1016/j.stemcr.2025.102675)
Supplement: Document S1. Methods S1 and S2 [file mmc1.pdf]

Stem Cell Reports, Volume *20*

Supplemental Information

The marketing of "stem cell" supplements on Amazon.com: Assessing alignment with regulatory frameworks in the United States and Canada

Alessandro R. Marcon, Marco Zenone, Vincenza Boniface, Sophie Sigfstead, Blake Murdoch, and Timothy Caulfield

Supplementary Materials

SM1: A critical overview of supplement regulation in Canada and the United States

The United States

In the United States, ingestible supplements (but not topical creams or sprays) are classified as dietary supplements, but unlike Canada, can gain market access without any significant regulatory oversight.[1-3] Proposals for tighter government regulation of supplements were considered in the early 1990s but ultimately quashed in part by industry pushback.[4] The finalized Dietary Supplement Health & Education Act (DSHEA) of 1994 does not obligate manufacturers to submit any premarket safety or efficacy data unless said product contains a "new dietary ingredient."[2,5] Dietary supplements therefore do not require a Federal Drug Administration (FDA) license for market access. As a result, the FDA is unaware when new products appear on the market nor does it have a list of all products currently available.[2]

Dietary supplement regulation in the US is a joint post-market focused effort between the FDA and the Federal Trade Commission.[6] It is industry's responsibility to ensure product safety and label accuracy alignment with FDA and DSHEA regulations prior to marketing. Once on the market, the FDA, in partnership with the FTC can pursue regulatory action for violations of ingredient or branding accurac.[2] Broadly the FDA regulates for product "safety, quality, and labeling" while the FTC regulates product advertising.[2,6] It is the responsibility of manufacturers and distributors to record, investigate and distribute reports of serious adverse events to products to the FDA.[2,6] The FDA uses this information as well consumer complaints, market monitoring, sample analyses, and other assessment/surveillance mechanisms to launch investigations.[2]

The regulation of product claims pertains to labels (FDA focused) and advertising discourse (FTC focused). These claims consist primarily of "health claims" (dietary impact on health ailment), "nutrient content claims" (nutrient levels), and "structure/function claims" (product affect on or maintenance of a body's structure or function).[2,5] Similar to "general health claims" in the Canadian regulatory framework (see below), "structure/function" claims, which are distinct from disease claims, cannot claim to "diagnose, mitigate, treat, cure, or prevent" disease but can use general terms such as "promote," "maintain," "support," "strengthen," "improve," and "protect" to discuss product function in relation to one's bodily mechanisms.[7,8] Additional claims can pertain to nutrient deficiencies or "general well-being." All claims are subject to accuracy and corresponding evidentiary-support requirements ensuring claims are "truthful," "not false" "and not misleading".[2,7,9] It the responsibility of industry to have evidence to substantiate claims, but the production of said evidence is initiated by the FDA.[2] To mediate this dynamic, when any product includes a claim in public discourse, said claim must, by law, include the disclaimer: "This statement has not been evaluated by the Food and Drug Administration. This product is not intended to diagnose, treat, cure, or prevent any disease".[2]

In 2022, the FTC published a "Health Products Compliance Guidance" document, which doesn't carry legal force or effect but offers explanations and interpretations of FTC advertising law.[6] It outlines how health product advertising pertains to all media, including events, online ads, social media and influencer marketing, and how all information in these sources "must comply with the same truth-in-advertising principles that apply to traditional ads."[6] Benefit and safety claim regulation applies to both direct, explicit claims but also indirect or implied impressions created by marketing discourse. The FTC guidelines foreground consumer interpretation, whereby the summative ad impressions are assessed, incorporating all reasonable consumer interpretations. Ad deception includes omission of important information disclosures, including limitations of health benefits. Claim substantiation and corresponding rigour is required to match the nature of the claim ensuring there is adequate scientific support.[6] The guide notes that for safety and efficacy claims, scientific evidence is required that is "competent," "reliable" and accurately reflective of "the entire body of evidence" needed to substantiate a representation's truth impressions.[6] It is therefore prohibited to give a misleading impression of scientific consensus, to rely on flawed, inadequate, or anecdotal-based studies, or to present consumer testimonials as evidence.[6]

Dietary supplement regulation in the US has been critiqued for offering limited consumer protection despite the FTC having settled or adjudicated over 200 cases "involving false or misleading advertising claims about the benefits or safety of dietary supplements or other health-related products." [1,4,6] Indeed, there are an estimated total of more than 100,000 products on the US market.[10] It has been argued that US dietary supplement regulation is primarily focused on enabling commerce while passing responsibility to consumers to investigate the verity, safety, and usefulness of marketed products.[1,2] The FDA assists with these consumer activities by producing supplement-focused educational materials[11] and consumer alerts, including for example, a 2020 alert on regenerative medicine products including stem cells and exosomes.[12] The question remains as to whether these government issues documents have any impact on consumer behaviour.

Canada

In Canada, most supplements are classified as natural health products (NHPs), including topical creams and sprays, and are regulated by Health Canada's Natural and Non-prescription Health Products Directorate (NNHPD) under the Food and Drugs Act (1985) and the Natural Health Products Regulations (2004). Prior to 2004, supplements in Canada could be classified as either food or drugs based on medicinal characteristics. In response to growing supplement use, coupled with public desire for increased regulation, an extensive nationwide consultation process resulted in the new regulatory framework that sought to balance consumer safety with consumer freedom by regulating the sale, manufacturing, distribution, and storage of NHPs.[2,8,13]

Unlike the United States, for NHPs to gain market access in Canada, manufacturers must obtain a product license, and corresponding manufacturing site license, contingent upon the provision of details of a product's ingredient, dose, potency, manufacturing site, and consumer use details. Additionally, information must be provided "that demonstrates the safety and efficacy of the natural health product when it is used in accordance with the

recommended conditions of use."[8] NHP regulation is distinct and separate from that of prescription drugs, aligning its practice to NHPs designated "lower risk nature."[3] As such, while NHP efficacy claims require substantiating evidence, health claims for licensing follow either a "modern" or "traditional" pathway, and the scientific rigour of evidential support aligns with the health claim severity in conjunction with a product's risk profile.[14] A high-level risk product pertains to more serious ("potentially life-threatening") health scenarios and/or narrower safety margins.[14] Corresponding health claims relate to diseases/conditions, categorized as "Serious", "Major," or Minor", and to health effects, categorized by product function related to diagnostics, treatment, cures, risk reduction, prevention, antioxidant qualities, or general health maintenance, support, and promotion.[14] Regardless of risk-level, further safety and efficacy evidence may be required for licensing products directed towards "vulnerable sub-populations," which includes pregnant people, the elderly, and children.[14]

Like "structure/function claims" in the United States, the category of "general health claims" in Canada has particular regulatory relevance for NHPs like stem cell supplements. "General health claims" are defined as having "low therapeutic impact," and relate to lower-risk health scenarios.[14] "General health claims" are defined and presented in a broader, less-precise manner thereby explicitly acknowledging a product's limited potential for significant health impact. There is a linguistic component to this categorization. Here, claims typically refer to a product's ability to "maintain," "promote," "support" [a beneficial health attribute], to "help" [address or prevent (one aspect of) an ailment], or to a product's "source of" [a beneficial ingredient].[14] "General health claims" are thus distinct from the concrete and causal defining language describing a products' ability to address the "mitigation, prevention, or cure of serious or major conditions".[14] Specifically, a product's "general health claims" cannot refer to the treatment or curing potential of Schedule A diseases listed in the Food and Drug Act but "may support mechanisms of action associated with reduction of the risk of a Schedule A disease."[14]

Considerable regulatory flexibility is therefore granted towards the substantiating evidence required for "general health claims." Evidence assessments for "general health claims," state that "general health claims must not be false or misleading and their accuracy must be established through an established methodology to meet the appropriate standard of evidence."[2] NHPs with "general health claims," however, can rely on supportive evidence where "there are challenges in determining the therapeutic effect."[2] These include "patterns of evidence" from multiple sources, "specific end-points," which focus on overall health/medicine system benefits, or "qualifications," which essentially relates to the hedging of health claims with grammar modality (e.g. "could," "likely," "may," etc.).[2] In sum, "general health claims," which include "general health maintenance claims," operate in an arguably ambiguous state of only suggestive or potential benefit, whether that benefit relates to a body's normal or ailment-suffering states.

The regulation of NHPs in Canada has faced scrutiny in both formal assessments and popular discourse.[3,4,13] Notably, the Office of the Auditor General's 2021 audit of Health Canada's NHP regulation program found that "Canada fell short of ensuring that products were safe and effective."[13] Issues were found in both approval and monitoring processes,

noting that “little” was done “to prevent poor information from being given to consumers about licensed natural health products.”[13] Additionally, analysis on a sample of licensed NHPs found 88% had been advertised with misleading label information, and 56% with misleading information related to health claims and consumer uses.[13] The report recommended improved monitoring and oversight mechanisms of NHP quality, labeling, and advertising and for strengthening compliance and enforcement tools. These recommendations were acknowledged and accepted by Health Canada.[3,13] In 2023, Health Canada extended the Protecting Canadians from Unsafe Drugs Act (Vanessa’s Law) to NHPs. This change increases Health Canada’s regulatory authority by granting it the ability to strengthen surveillance capacity, recall unsafe products, impose greater financial penalties, and mandate further product testing from manufacturers.[15] Furthermore, Health Canada is currently working towards improving NHP labelling requirements and instilling fees for pre-market evaluation, site licences, and rights to sell.[15,16]

References

1. Binns CW, Lee MK, Lee AH. Problems and Prospects: Public Health Regulation of Dietary Supplements. Annual Review of Public Health. 2018 Apr;39(1):403–20.

2. U.S. Food and Drug Administration. Questions and Answers on Dietary Supplements [Internet]. U.S. Food and Drug Administration; 2022 [cited 2024 Sep 5]. Available from: https://www.fda.gov/food/information-consumers-using-dietary-supplements/questions-and-answers-dietary-supplements

3. Health Canada. About Natural Health Product Regulation in Canada [Internet]. Canada.ca. Government of Canada; 2004 [cited 2024 Sep 5]. Available from: https://www.canada.ca/en/health-canada/services/drugs-health-products/natural-non-prescription/regulation.html

4. Jarry J. Office for Science and Society. 2023 [cited 2024 Sep 5]. The False Reassurance of Dietary Supplement Regulation. Available from: https://www.mcgill.ca/oss/article/critical-thinking-health-and-nutrition/false-reassurance-dietary-supplement-regulation

5. Office of Dietary Supplements. Dietary Supplement Health and Education Act of 1994 [Internet]. U.S. Department of Health & Human Services; 1994 [cited 2024 Oct 3]. Available from: https://ods.od.nih.gov/About/DSHEA_Wording.aspx

6. Federal Trade Commission. Health Products Compliance Guidance [Internet]. Federal Trade Commission. [cited 2024 Oct 3] Available from: https://www.ftc.gov/system/files/ftc_gov/pdf/Health-Products-Compliance-Guidance.pdf

7. U.S. Food and Drug Administration. Guidance for Industry: Substantiation for Dietary Supplement Claims Made Under Section 403(r) (6) of the Federal Food, Drug, and Cosmetic Act [Internet]. U.S. Food and Drug Administration Center for Food Safety and Applied

Nutrition; 2022. Available from: https://www.fda.gov/regulatory-information/search-fda-guidance-documents/guidance-industry-substantiation-dietary-supplement-claims-made-under-section-403r-6-federal-food

8. Department of Justice. Canada. Natural Health Products Regulations [Internet]. Minister of Justice; 2023 [cited 2024 Jun 14]. Available from: https://laws-lois.justice.gc.ca/PDF/SOR-2003-196.pdf

9. U.S. Food and Drug Administration. Structure/Function Claims [Internet]. U.S. Food and Drug Administration - Human Foods Program; 2024 [cited 2024 Sep 5]. Available from: https://www.fda.gov/food/nutrition-food-labeling-and-critical-foods/structurefunction-claims

10. U.S. Food and Drug Administration. FDA's Regulation of Dietary Supplements with Dr. Cara Welch [Internet]. FDA - Center for Drug Evaluation and Research; 2023 Dec 13 [cited 2024 Oct 2]; Available from: https://www.fda.gov/drugs/news-events-human-drugs/fdas-regulation-dietary-supplements-dr-cara-welch

11. U.S. Food and Drug Administration. FDA Launches New Dietary Supplement Education Initiative[Internet]. FDA - Center for Food Safety and Applied Nutrition; 2022 Jun 2 [cited 2024 Sep 5]; Available from: https://www.fda.gov/food/cfsan-constituent-updates/fda-launches-new-dietary-supplement-education-initiative

12. U.S. Food and Drug Administration. Consumer Alert on Regenerative Medicine Products Including Stem Cells and Exosomes [Internet]. FDA - Center for Biologics Evaluation and Research; 2020 Jul 22 [cited 2024 Sep 5]; Available from: https://www.fda.gov/vaccines-blood-biologics/consumers-biologics/consumer-alert-regenerative-medicine-products-including-stem-cells-and-exosomes

13. Health Canada. Report 2—Natural Health Products—Health Canada [Internet]. Government of Canada - Office of the Auditor General of Canada; 2021 [cited 2024 Sep 5]. Available from: https://www.oag-bvg.gc.ca/internet/English/parl_cesd_202104_02_e_43806.html

14. Health Canada. Pathway for Licensing Natural Health Products Making Modern Health Claims [Internet]. Health Canada; 2012 [cited 2024 Sep 5]. Available from: https://www.canada.ca/en/health-canada/services/drugs-health-products/natural-non-prescription/legislation-guidelines/guidance-documents/pathway-licensing-making-modern-health-claims.html

15. Health Canada. Protecting Canadians from Unsafe Drugs Act (Vanessa's Law) Amendments to the Food and Drugs Act (Bill C-17) [Internet]. www.canada.ca. Health Canada; 2013 [cited 2024 Sep 5]. Available from: https://www.canada.ca/en/health-canada/services/drugs-health-products/legislation-guidelines/protecting-canadians-unsafe-drugs-act-vanessa-law-amendments-food-drugs-act.html

16. Health Canada. Natural Health product regulation in Canada: Natural health product cost recovery Overview [Internet]. Health Canada;2023 [cited 2024 Sep 12]. Available from: https://www.canada.ca/en/health-canada/services/drugs-health-products/natural-non-prescription/regulation/cost-recovery.html

Supplementary Materials 2: Methods (detailed description)

On December 22, 2023, we searched "stem cells" on Amazon.com for all products, both sponsored and non-sponsored, listed on in the category "Health & Household" and sub-category "Vitamins, Minerals, and Supplements." This search returned 991 total product URLS, and all corresponding metadata: product name, company, number of ratings, rating score (average), price, format, and sponsored/not-sponsored designation. All non-functional URLs were removed as well as product duplicates, such as the same product appearing in different sizes (e.g. 3 pack, 6 pack, large bottle small bottle, etc.). Next, each unique product ad was verified to contain either an explicit mention of stem cells or stem cell adjacent language in relation to cells or cell activity (e.g. "regeneration," "rejuvenation," "renewal," "repair" etc.) Products that included neither explicit or adjacent stem cell language were excluded.

The finalized data set consisted of (N=184) stem cell supplements (170 (92.4%) non-sponsored) from 133 unique companies. Content analysis was performed on all remaining products, and applied to information appearing anywhere in an ad, including images. Prior to engaging the data, the content analysis approached was directed towards a focus on health claims, promotional product descriptors, and appeals to science/scientific evidence. As detailed in the introduction, there is a relationship between health claims and the objective of and requirement for supporting claims with scientific evidence. The first stage of coding included determining whether the products were currently available, available in Canada, and whether they included a defining description of stem cells. Products not "currently available" were included in the analysis as none of these products were listed as discontinued by the manufacturer. There was no indication as to when the products became unavailable or if their availability status was final. Next, analysis captured which health ailments the products claimed to address, which beneficial descriptors were used to describe products, whether science rhetoric or scientific evidence was used to demonstrate quality/efficacy, and whether health care practitioners, including health scientists were mentioned or appeared in images. Only explicit mentions (either in text or images) were included as substantiating evidence for each category, thus reducing all subjective interpretation. For example, in the category of appeals to scientific evidence, only direct mentions of, for example, "science," "scientists," "trials," or "research" were coded as present. More subjective interpretations of science-related images (e.g. DNA helices; space imagery, futuristic graphics) were not included. Explicit mentions of healthcare practitioners (e.g. "doctors," "physicians," "nurses," etc.) were included as were images of individuals wearing, for example, lab coats and stethoscopes. Two coders coded the dataset over a two-week period and met periodically to ensure there was no coding ambiguity.

Following coding completion, a third coder checked all coding, observing 90% accuracy. Additional findings observed by the third coder were verified and incorporated.
