# Supplementary material for: The marketing of “stem cell” supplements on Amazon.com: Assessing alignment with regulatory frameworks in the United States and Canada
Source: Stem Cell Reports. 2025 Oct 9;20(11):102675. doi: 10.1016/j.stemcr.2025.102675 (PMC12790744; doi:10.1016/j.stemcr.2025.102675)
Supplement: Document S2. Article plus supplemental information [file mmc3.pdf]

Stem Cell Reports
Forum
OPEN ACCESS

The marketing of "stem cell" supplements on Amazon.com: Assessing alignment with regulatory frameworks in the United States and Canada

Alessandro R. Marcon,[1,*] Marco Zenone,[1,2] Vincenza Boniface,[1] Sophie Sigfstead,[1] Blake Murdoch,[1] and Timothy Caulfield[1]
[1]Health Law Institute, Office 470, Faculty of Law, University of Alberta, Edmonton, AB T6G 2H5, Canada
[2]Faculty of Health Sciences, University of Ottawa, Ottawa, ON K1H 8M5, Canada
*Correspondence: marcon@ualberta.ca
https://doi.org/10.1016/j.stemcr.2025.102675

The direct-to-consumer marketing of stem cell interventions now includes an emerging ecommerce marketplace of "stem cell" supplements. This research assessed the marketing of stem cell supplements on Amazon.com, evaluating how that marketing aligned or conflicted with regulatory frameworks in the United States and Canada. Given the results, new regulation strategies should be considered to offer greater transparency and consumer protection.

INTRODUCTION

Scientifically unsupported commercial activity has long accompanied developments in stem cell research. Clinics' online marketing of stem cell treatments for illness, pain, repair, and injury has been studied extensively worldwide, consistently reporting unproven and inflated health benefit claims (Lyons et al., 2021; Turner et al., 2024). Discourse in, but not limited to, these commercial spaces often distorts, exaggerates, and manipulates scientific evidence and rhetoric to sell stem-cell products, therapies, and ideas (Turner et al., 2024; Ellythy et al., 2025)—a phenomenon called science hype or "scienceploitation" (Caulfield et al., 2019). Misrepresenting scientific legitimacy breaches ethical guidelines and exploits consumers. In response, calls exist for increased regulation, including from the International Society for Stem Cell Research (ISSCR) calling for "accurate, current, balanced, and responsive public representations of stem cell research" (ISSCR, 2021). Effective regulation has been challenging, however, as online cross-border marketing can evolve quickly, challenge localized regulatory frameworks, and present with opaque platform mechanisms (Lyons et al., 2021; Turner et al., 2024), such as the obfuscation of paid-promotion content and non-transparent black box algorithms governing platform activity.

The marketplace of stem cell therapies and products continues to evolve. Recently, "stem cell" supplements have emerged in online marketplaces. Supplements remain popular in North America and abroad (Knoepfler, 2024; Sullivan, 2025). Consisting typically of pills, powders, or liquids, supplements are promoted to augment one's diet with nutrients such as herbs, probiotics, amino acids, enzymes, vitamins, or minerals. They form a core component of the highly profitable wellness industry, where individual health is promoted as an individual responsibility to achieve ever-shifting ideas of optimization (Derkatch, 2022). In these contexts, wellness advocates promoting supplements commonly view regulatory authorities as restricting autonomous health promotion and contributing to society's toxicity, in large part by enabling the pharmaceutical industry (Derkatch, 2022). Despite sharing industry connections, supplements, in contrast to pharmaceuticals, are commonly viewed among wellness advocates as natural, pure, and safe (Derkatch, 2022). Supplements, however, may suffer from labeling inaccuracies, offer few health benefits, save nutrient deficiency cases, and lead to health risks (Binns et al., 2018).

Consumer desire for, and profitability of, supplements creates complex challenges for regulatory bodies (Sullivan, 2025; Derkatch, 2022; Jarry, 2023). In Canada and the United States, where supplements are classified as Natural Health Products (NHPs) and dietary supplements, respectively, regulatory efforts have been criticized for ineffectiveness while also being met with resistance from industry and the public (Sullivan, 2025; Derkatch, 2022; Jarry, 2023). Currently, dietary supplements are not subjected to regulatory approval—in terms of safety or efficacy—before gaining market access in the United States. In contrast, market entry for NHPs in Canada requires product and manufacturing site licenses from Health Canada (see supplemental information [SM 1] for detailed comparative analysis of both frameworks) (Health Canada, 2012; U.S. Food and Drug Administration, 2024; US FTC, 2024). Aside from this key difference, both overarching regulatory parameters are similar (Jarry, 2023). In both jurisdictions, industry is prohibited from making strong causal claims around a products' ability to diagnose, prevent, cure, or mitigate serious ailments and disease (Health Canada, 2012; U.S. Food and Drug Administration, 2024; US FTC, 2024). In the United States, all dietary supplements marketed with a health claim are required to include the following disclaimer: "This statement has not been evaluated by the Food and Drug Administration. This product is not intended to diagnose, treat,

Published by Elsevier Inc. on behalf of International Society for Stem Cell Research.
This is an open access article under the CC BY license (http://creativecommons.org/licenses/by/4.0/).

cure, or prevent any disease" (U.S. Food and Drug Administration, 2024).

Similar regulatory measures in both jurisdictions detail how a product's health claims need substantiation with sufficient evidence and must not be marketed by distorting the scientific evidence base (Health Canada, 2012; U.S. Food and Drug Administration, 2024; US FTC, 2024). Products that claim greater efficacy, specifically in more serious health contexts, would—as described—require a stronger evidentiary base. However, while both jurisdictions present consumer-protecting regulatory measures against misleading claims, both permit industry to market products' health claims using vague, indirect, or hedged language, via "structure/function" claims in the United States and "general health claims" in Canada (SM 1). Both claim types broadly apply to lower risk health applications and permit a product's mechanism of action to be described with non-direct verbs (e.g., "promote," "maintain," "support," and "help") or with causality expressed with modality (e.g., "may," "could," and "might"). Product marketing is thus permissible if it is only suggestive of potential benefit and if the relevant substantiating scientific evidence-base consensus is not presented misleadingly (SM 1).

Supplement regulatory breaches have been observed in both Canada and the United States. In the US, the Federal Trade Commission (FTC), which coordinates with the Food and Drug Administration (FDA) but addresses advertising discourse, has settled or adjudicated over 200 cases "involving false or misleading advertising claims about the benefits or safety of dietary supplements or other health-related products" (US FTC, 2024). In Canada, the Office of the Auditor General's audit of Health Canada's NHP regulation program found that "Canada fell short of ensuring that products were safe and effective" (Office of the Auditor General of Canada, 2021). Issues were found in both approval and monitoring processes, noting that "little" was done "to prevent poor information from being given to consumers about licensed natural health products" (Office of the Auditor General of Canada, 2021). Additionally, analysis on a sample of licensed NHPs found misleading information in 88% of products' labels and 56% of product's health claims and consumer uses (Office of the Auditor General of Canada, 2021).

Funded to investigate emerging trends in regenerative medicine commercialization, we assessed these regulatory frameworks in the context of emerging stem cell supplements. As the placement and storage of human stem cells in pills, liquids, or capsules is scientifically implausible, we sought to investigate how stem cell supplements were defined and promoted in popular online marketplaces. In December 2023, we built a dataset of all stem cell products found on the ecommerce platform Amazon.com in the category "Vitamins, Minerals, and Supplements." Our finalized dataset of 184 stem cell supplement listings from 133 companies included only unique (non-duplicated) products with a described stem cell-related function. Coding captured health ailments addressed by products and corresponding rhetoric in health claims, use of beneficial product descriptors, and the presence of scientific rhetoric as well as health care practitioners/scientists (see SM 2 for a complete methods overview). Capturing the presence of scientific rhetoric/evidence and health care practitioners (i.e., appeals to scientific expertise) was included as a metric for assessing science hype or scienceploitation (Caulfield et al., 2019). Stratified descriptive statistical analysis was performed between products available in Canada and the United States versus only in the United States.

HOW STEM CELL SUPPLEMENTS WERE MARKETED ON AMAZON.COM

The stem cell supplement advertisements on Amazon.com (n = 184) (Table S1) claimed that products either contained stem cells, created more stem cells, or improved stem cell functioning. These ads sometimes defined stem cells or described their functionality (61, 33.2%) but almost always made health claims in relation to particular health ailments (173, 94.0%). Listings commonly made claims with regard to multiple health ailments/contexts (Table 1). Beneficial product descriptors were listed in the majority of products (155, 84.2%), and over 40% of all products made an explicit mention of science or scientific evidence in relation to product quality or efficacy (e.g., "cutting-edge science," "clinically tested," or "scientifically proven") (77, 41.8%). Health care professionals had a smaller but not negligible presence (65, 35.3%) (Table 1).

Marketing portrayed beneficial effect for over 60 diverse ailments, most frequently regarding aging, immunity, energy, healing/repair, skin health, overall/general health, brain health, and cardiovascular functioning (Table 1). All health claims were expressed with vague, noncausal verbs such as "supports," "promotes," "bolsters," "protects," etc., alongside the use of asterisk-marked hedging statements, and modal verbs such as "may," "could," or "can." The required FDA dietary supplement disclaimer appeared in all dietary supplement ads with a health claim. For beneficial product descriptors (155, 84.2%), products on average listed 2.5 characteristics indicative of wellness trends, the most used being

Table 1. "Stem cell" supplement information contained in Amazon.com listings (N = 184)

Information in product listing	Products available in US and Canada	Products available only in US	All products
Listed products[a]	**89 (48.4%)**	**95 (51.6%)**	**184 (100%)**
Defining characteristics of stem cells[b]	32 (36.0%)	29 (30.5%)	61 (33.2%)
Benefits listed for particular health ailments	84 (94.4%)	89 (93.7%)	173 (94.0%)
Mean average of health ailments per listing	4.6	5.1	4.8
Ads with ≥10 ailments	4 (4.5%)	9 (9.5%)	13 (7.1%)
≥5 ailments	45 (50.6%)	51 (53.6%)	96 (52.2%)
≥3 ailments	68 (76.4%)	73 (76.8%)	141 (76.6%)
Only 1 ailment	6 (6.7%)	10 (10.5%)	16 (8.7%)
Total number of health ailments	50	52	64
Health ailments listed by ranking (min 7% of total)			
Aging (anti-aging/healthy)	56 (62.9%)	47 (49.5%)	103 (56.0%)
Immunity	30 (33.7%)	47 (49.5%)	77 (41.8%)
Energy	36 (40.4%)	31 (32.6%)	67 (36.4%)
Healing/repair	31 (34.8%)	30 (31.6%)	61 (33.2%)
Skin	26 (29.2%)	26 (27.4%)	52 (28.3%)
Brain-related (e.g., cognitive functions, "brain fog," "thinking," and "focus")	26 (29.2%)	20 (21.1%)	46 (25.0%)
Overall/general health	20 (22.5%)	25 (26.3%)	45 (24.5%)
Cardiovascular related	18 (20.2%)	23 (24.2%)	41 (22.3%)
Joints	12 (13.5%)	21 (22.5%)	33 (17.9%)
Antioxidant related	15 (16.9%)	16 (16.8%)	31 (16.8%)
Bones	6 (6.7%)	18 (18.9%)	24 (13.0%)
Muscles	11 (12.4%)	11 (11.6%)	22 (12.0%)
Inflammation	9 (10.1%)	12 (12.6%)	21 (11.4%)
Metabolism	12 (13.5%)	9 (9.5%)	21 (11.4%)
Hair	8 (9.0%)	10 (10.5%)	18 (9.8%)
Heart specific	8 (9.0%)	9 (9.5%)	17 (9.2%)
Memory specific	8 (9.0%)	8 (8.4%)	16 (8.7%)
Mood	4 (4.5%)	11 (11.6%)	15 (8.2%)
Stress	8 (9.0%)	5 (5.3%)	13 (7.1%)

(*Continued on next page*)

"natural" (103, 56.0%), GMO-free (75, 40.8%), gluten-free (61, 33.2%), and pure/organic (52, 28.3%) (Table 1). No considerable trends were observed between products with differing availability between Canada and the United States (Table 1).

REGULATORY CHALLENGES AND STRENGTHENING STRATEGIES

The marketing of stem cell supplements on Amazon.com highlights regulatory limitations and challenges. This stem cell supplement marketing did not explicitly mention serious, life-threatening diseases (e.g., cancer, asthma, arthritis, dementia, congestive heart failure, etc.) or use direct causal language of effect (e.g., "mitigate," "prevent," "cure," or "treat"). Products were thus arguably aligned with the "general health claims" and "structure/function claims" in the respective Canadian and American regulatory frameworks. The current frameworks therefore permit and arguably enable free rein for vague (and scientifically unsupported) claims for general health ailments and also more serious ailments presented with discreet language.

There is a problematic and potentially dangerous assumption that consumers accurately interpret this marketing as only claiming minor therapeutic potential for non-serious conditions. For example, although "dementia" was absent in the marketing, anti-aging, memory, and cognitive function ailments were frequently mentioned. Explicit mentioning of disease prevention was absent, but text with "strengthening," "supporting," or "promoting" immunity was common. Further, this marketing arguably generates consumer impressions of ample scientific evidentiary support, at odds with the current state of stem cell therapeutics (Knoepfler, 2024). As detailed in the FTC's 2022 "Health

Table 1. ***Continued***

Information in product listing	Products available in US and Canada	Products available only in US	All products
Detailing of scientific support for quality/efficacy	**41 (46.1%)**	**36 (37.9%)**	**77 (41.8%)**
Inclusion of health care/health science professionals	**33 (37.1%)**	**32 (33.7%)**	**65 (35.3%)**
Beneficial product descriptors (min 6% of total)	**71 (79.8%)**	**84 (88.5%)**	**155 (84.2%)**
Natural	42 (47.2%)	61 (64.2%)	103 (56.0%)
GMO-free	32 (36.0%)	43 (45.3%)	75 (40.8%)
Gluten-free	31 (34.8%)	30 (31.6%)	61 (33.2%)
Pure/organic	28 (31.5%)	24 (25.3%)	52 (28.3%)
Vegan/vegetarian	21 (23.6%)	27 (28.4%)	48 (26.1%)
No fillers/preservatives	24 (27.0%)	21 (22.7%)	45 (24.5%)
Soy-free	12 (13.5%)	10 (10.4%)	22 (12.0%)
Ancestral/traditional product	5 (5.6%)	14 (14.7%)	19 (10.3%)
Toxin-free	4 (4.5%)	8 (8.4%)	12 (6.5%)

[a]Products listed as not currently available, not discontinued by the manufacturer, were included in the dataset as unavailability could relate to a lack of current supply in the process of being restocked.
[b]Products defining stem cells and/or describing their function in human anatomy.

Products Compliance Guidance" document, which does not carry legal force or effect but offers explanations and interpretations of FTC advertising law, there is a regulatory need to interpret marketing discourse through reasonable consumer interpretations of an entire ad's impression (US FTC, 2024). It stresses that it is prohibited for ads to give a misleading impression of scientific consensus (US FTC, 2024). There were no clear marketing differences between products available in Canada versus the United States. This is unsurprising, given the similarity of regulatory platforms aside from Canada's unique licensing mandates. There are numerous reasons a company may not pursue Canadian markets, and future research could assess the potential impact of Canada's licensing requirements.

Whether aligning with current regulations permitting vague marketing or conflicting with regulation prohibiting a distortion of the evidentiary base, stem cell supplements with no clear consumer benefit are being promoted and sold on Amazon.com. The question stands whether—and which kinds of—regulatory reform is needed in response. Supplement regulation in North America has been (ardently) resisted in the past (Derkatch, 2022; Binns et al., 2018; Jarry, 2023), and efforts to frame supplements as distinct from the perceived harms and dangers of "mainstream" pharmaceuticals (Derkatch, 2022) were observed in the marketing of stem cell supplements on Amazon.com (82% included common wellness characteristics, such as "naturalness" [56%]). Given historical efforts to restrict supplement access, there is good reason to believe that increasing regulation dramatically could face backlash from consumers and industry.

Regulatory frameworks, however, should not enable manufacturers to make inaccurate, unsubstantiated product claims. Health Canada has acknowledged its NHP regulatory limitations and is implementing changes to strengthen capacity (Office of the Auditor General of Canada, 2021). Notably, changes will increase licensing costs, helping reduce the financial burden on Health Canada, which may deter frivolous and egregiously inadequate applications. Tightening market access may lead to fewer examples of highly problematic marketing and facilitate post-market surveillance, including advertising with health claims and appeals to scientific efficacy. It would be a dramatic policy shift to regulate market-entry access (e.g., with licensing requirements) in the United States, yet doing so could enhance consumer safeguards.

Monitoring products' marketing is labor- and time intensive. While a few hundred cases of supplement regulation have occurred in the United States, more than 100,000 dietary supplements are estimated to be available for consumers (Sullivan, 2025). The stem cell products observed in this study may not be classified as high risk, thus falling outside the urgency scope of regulators. And yet, a failure to regulate in any meaningful way hurts consumers by allowing the misleading marketing of deceptive products. It hinders understandings of, and trust in, stem cell science and related efforts for evidence-based policy making. Research shows that increased regulation of stem cell commercialization can offer increased consumer protections (Ikonomou et al., 2024). Approaches and mechanisms to surveil online marketing could be created with input from existing guidelines (ISSCR, 2021) that identify keywords associated with serious health contexts or vulnerable populations (e.g., the elderly or pregnant people). Uses of scientific rhetoric claiming evidentiary support—examples of which are described in this research—could trigger increased investigation, including requirements to produce corresponding evidence.

Though complex, governments could begin working with automated AI systems to flag marketing cases for further investigation. Regulatory bodies must be sufficiently funded and mandated to regulate on the basis of scientific accuracies. Given the expanding market for supplements and wellness products—and the growing evidence of deceptive practices and potential harms—the creation of new regulatory strategies seems essential to strengthen consumers' ability to make evidence-informed decisions.

ACKNOWLEDGMENTS

This research was funded by the Stem Cell Network (grant #917390) under the project title: Law, Public Policy and Social License for Next-Generation Regenerative Medicine. This funding agency played no role in any aspect of this research including the decision for publication. No authors were precluded from accessing data in the study, and all authors accept responsibility to submit the manuscript for publication. Authors wish to thank Robyn Hyde-Lay for managerial assistance with this research.

AUTHOR CONTRIBUTIONS

A.R.M., M.Z., and T.C. designed the study. M.Z., A.R.M., and V.B. collected, organized, and managed data. A.R.M., M.Z., V.B., and S.S. analyzed and interpreted the data with input from B.M. and T.C. A.R.M. wrote the first draft of the manuscript. M.Z., V.B., S.S., B.M., and T.C. reviewed and edited the manuscript. All authors had access to the data throughout the research. A.R.M., M.Z., V.B., and S.S. verified the data reported in the manuscript.

DECLARATION OF INTERESTS

The authors declare no competing interests.

SUPPLEMENTAL INFORMATION

Supplemental information can be found online at https://doi.org/10.1016/j.stemcr.2025.102675.

REFERENCES

Binns, C.W., Lee, M.K., and Lee, A.H. (2018). Problems and Prospects: Public Health Regulation of Dietary Supplements. Annu. Rev. Public Health *39*, 403–420.

Caulfield, T., Marcon, A.R., Murdoch, B., Brown, J.M., Perrault, S.T., Jarry, J., Snyder, J., Anthony, S.J., Brooks, S., Master, Z., et al. (2019). Health misinformation and the power of narrative messaging in the public sphere. Bioethics *2*, 52–60.

Derkatch, C. (2022). Why Wellness Sells: Natural Health in a Pharmaceutical Culture (JHU Press).

Ellythy, L., Addani, M., and Master, Z. (2025). An analysis of stem cell training programs for physicians in the US—A mirage of credibility. Stem Cell Rep. *20*, 102510.

Federal Trade Commission. Health Products Compliance Guidance [Internet]. Federal Trade Commission. [cited 2024 Sep 5]. Available from: https://www.ftc.gov/system/files/ftc_gov/pdf/Health-Products-Compliance-Guidance.pdf.

Health Canada. Pathway for Licensing Natural Health Products Making Modern Health Claims [Internet]. Health Canada; 2012 [cited 2024 Sep 5]. Available from: https://www.canada.ca/en/health-canada/services/drugs-health-products/natural-non-prescription/legislation-guidelines/guidance-documents/pathway-licensing-making-modern-health-claims.html.

Ikonomou, L., Munsie, M., Power, C., Sipp, D., Turner, L., and Rasko, J.E.J. (2024). Effective regulatory responses to predatory stem cell markets in Australia and Canada. Cell Stem Cell *31*, 1393–1397.

ISSCR (2021). Guidelines for Stem Cell Research and Clinical Translation [Internet] (International Society for Stem Cell Research). https://www.isscr.org/guidelines.

Jarry J. Office for Science and Society. 2023 [cited 2024 Sep 5]. The False Reassurance of Dietary Supplement Regulation. Available from: https://www.mcgill.ca/oss/article/critical-thinking-health-and-nutrition/false-reassurance-dietary-supplement-regulation.

Knoepfler P. New review of stem cell supplements and pills [Internet]. The Niche. 2024 [cited 2024 Oct 2]. Available from: https://ipscell.com/2024/09/new-review-of-stem-cell-supplements-and-pills/.

Lyons, S., Salgaonkar, S., and Flaherty, G.T. (2021). International stem cell tourism: a critical literature review and evidence-based recommendations. Int. Health *14*, 132–141.

Office of the Auditor General of Canada (2021). Report 2—Natural Health Products—Health Canada [Internet]. https://www.oag-bvg.gc.ca/internet/English/parl_cesd_202104_02_e_43806.html.

Sullivan, K. Nutritional supplements and vitamins are more popular than ever. Do they work? NBC News. 2025 [cited 2025 Jul 7]. Available from: https://www.nbcnews.com/health/health-news/do-nutritional-supplements-really-work-regulation-rcna186045.

Turner, L., Wang, J.C., Martinez, J.R., Najjar, S., Rajapaksha Arachchilage, T., and Sahrai, V. (2024). US businesses engaged in direct-to-consumer marketing of perinatal stem cell interventions following the Food and Drug Administration's enforcement discretion era. Cytotherapy *26*, 393–403.

U.S. Food and Drug Administration. Structure/Function Claims [Internet]. U.S. Food and Drug Administration - Human Foods Program; 2024 [cited 2024 Sep 5]. Available from: https://www.fda.gov/food/nutrition-food-labeling-and-critical-foods/structurefunction-claims.

Stem Cell Reports, Volume *20*

Supplemental Information

The marketing of “stem cell” supplements on Amazon.com: Assessing alignment with regulatory frameworks in the United States and Canada

Alessandro R. Marcon, Marco Zenone, Vincenza Boniface, Sophie Sigfstead, Blake Murdoch, and Timothy Caulfield

Supplementary Materials

SM1: A critical overview of supplement regulation in Canada and the United States

The United States

In the United States, ingestible supplements (but not topical creams or sprays) are classified as dietary supplements, but unlike Canada, can gain market access without any significant regulatory oversight.[1-3] Proposals for tighter government regulation of supplements were considered in the early 1990s but ultimately quashed in part by industry pushback.[4] The finalized Dietary Supplement Health & Education Act (DSHEA) of 1994 does not obligate manufacturers to submit any premarket safety or efficacy data unless said product contains a "new dietary ingredient."[2,5] Dietary supplements therefore do not require a Federal Drug Administration (FDA) license for market access. As a result, the FDA is unaware when new products appear on the market nor does it have a list of all products currently available.[2]

Dietary supplement regulation in the US is a joint post-market focused effort between the FDA and the Federal Trade Commission.[6] It is industry's responsibility to ensure product safety and label accuracy alignment with FDA and DSHEA regulations prior to marketing. Once on the market, the FDA, in partnership with the FTC can pursue regulatory action for violations of ingredient or branding accurac.[2] Broadly the FDA regulates for product "safety, quality, and labeling" while the FTC regulates product advertising.[2,6] It is the responsibility of manufacturers and distributors to record, investigate and distribute reports of serious adverse events to products to the FDA.[2,6] The FDA uses this information as well consumer complaints, market monitoring, sample analyses, and other assessment/surveillance mechanisms to launch investigations.[2]

The regulation of product claims pertains to labels (FDA focused) and advertising discourse (FTC focused). These claims consist primarily of "health claims" (dietary impact on health ailment), "nutrient content claims" (nutrient levels), and "structure/function claims" (product affect on or maintenance of a body's structure or function).[2,5] Similar to "general health claims" in the Canadian regulatory framework (see below), "structure/function" claims, which are distinct from disease claims, cannot claim to "diagnose, mitigate, treat, cure, or prevent" disease but can use general terms such as "promote," "maintain," "support," "strengthen," "improve," and "protect" to discuss product function in relation to one's bodily mechanisms.[7,8] Additional claims can pertain to nutrient deficiencies or "general well-being." All claims are subject to accuracy and corresponding evidentiary-support requirements ensuring claims are "truthful," "not false" "and not misleading".[2,7,9] It the responsibility of industry to have evidence to substantiate claims, but the production of said evidence is initiated by the FDA.[2] To mediate this dynamic, when any product includes a claim in public discourse, said claim must, by law, include the disclaimer: "This statement has not been evaluated by the Food and Drug Administration. This product is not intended to diagnose, treat, cure, or prevent any disease".[2]

In 2022, the FTC published a "Health Products Compliance Guidance" document, which doesn't carry legal force or effect but offers explanations and interpretations of FTC advertising law.[6] It outlines how health product advertising pertains to all media, including events, online ads, social media and influencer marketing, and how all information in these sources "must comply with the same truth-in-advertising principles that apply to traditional ads."[6] Benefit and safety claim regulation applies to both direct, explicit claims but also indirect or implied impressions created by marketing discourse. The FTC guidelines foreground consumer interpretation, whereby the summative ad impressions are assessed, incorporating all reasonable consumer interpretations. Ad deception includes omission of important information disclosures, including limitations of health benefits. Claim substantiation and corresponding rigour is required to match the nature of the claim ensuring there is adequate scientific support.[6] The guide notes that for safety and efficacy claims, scientific evidence is required that is "competent," "reliable" and accurately reflective of "the entire body of evidence" needed to substantiate a representation's truth impressions.[6] It is therefore prohibited to give a misleading impression of scientific consensus, to rely on flawed, inadequate, or anecdotal-based studies, or to present consumer testimonials as evidence.[6]

Dietary supplement regulation in the US has been critiqued for offering limited consumer protection despite the FTC having settled or adjudicated over 200 cases "involving false or misleading advertising claims about the benefits or safety of dietary supplements or other health-related products." [1,4,6] Indeed, there are an estimated total of more than 100,000 products on the US market.[10] It has been argued that US dietary supplement regulation is primarily focused on enabling commerce while passing responsibility to consumers to investigate the verity, safety, and usefulness of marketed products.[1,2] The FDA assists with these consumer activities by producing supplement-focused educational materials[11] and consumer alerts, including for example, a 2020 alert on regenerative medicine products including stem cells and exosomes.[12] The question remains as to whether these government issues documents have any impact on consumer behaviour.

Canada

In Canada, most supplements are classified as natural health products (NHPs), including topical creams and sprays, and are regulated by Health Canada's Natural and Non-prescription Health Products Directorate (NNHPD) under the Food and Drugs Act (1985) and the Natural Health Products Regulations (2004). Prior to 2004, supplements in Canada could be classified as either food or drugs based on medicinal characteristics. In response to growing supplement use, coupled with public desire for increased regulation, an extensive nationwide consultation process resulted in the new regulatory framework that sought to balance consumer safety with consumer freedom by regulating the sale, manufacturing, distribution, and storage of NHPs.[2,8,13]

Unlike the United States, for NHPs to gain market access in Canada, manufacturers must obtain a product license, and corresponding manufacturing site license, contingent upon the provision of details of a product's ingredient, dose, potency, manufacturing site, and consumer use details. Additionally, information must be provided "that demonstrates the safety and efficacy of the natural health product when it is used in accordance with the

recommended conditions of use."[8] NHP regulation is distinct and separate from that of prescription drugs, aligning its practice to NHPs designated "lower risk nature."[3] As such, while NHP efficacy claims require substantiating evidence, health claims for licensing follow either a "modern" or "traditional" pathway, and the scientific rigour of evidential support aligns with the health claim severity in conjunction with a product's risk profile.[14] A high-level risk product pertains to more serious ("potentially life-threatening") health scenarios and/or narrower safety margins.[14] Corresponding health claims relate to diseases/conditions, categorized as "Serious", "Major," or Minor", and to health effects, categorized by product function related to diagnostics, treatment, cures, risk reduction, prevention, antioxidant qualities, or general health maintenance, support, and promotion.[14] Regardless of risk-level, further safety and efficacy evidence may be required for licensing products directed towards "vulnerable sub-populations," which includes pregnant people, the elderly, and children.[14]

Like "structure/function claims" in the United States, the category of "general health claims" in Canada has particular regulatory relevance for NHPs like stem cell supplements. "General health claims" are defined as having "low therapeutic impact," and relate to lower-risk health scenarios.[14] "General health claims" are defined and presented in a broader, less-precise manner thereby explicitly acknowledging a product's limited potential for significant health impact. There is a linguistic component to this categorization. Here, claims typically refer to a product's ability to "maintain," "promote," "support" [a beneficial health attribute], to "help" [address or prevent (one aspect of) an ailment], or to a product's "source of" [a beneficial ingredient].[14] "General health claims" are thus distinct from the concrete and causal defining language describing a products' ability to address the "mitigation, prevention, or cure of serious or major conditions".[14] Specifically, a product's "general health claims" cannot refer to the treatment or curing potential of Schedule A diseases listed in the Food and Drug Act but "may support mechanisms of action associated with reduction of the risk of a Schedule A disease."[14]

Considerable regulatory flexibility is therefore granted towards the substantiating evidence required for "general health claims." Evidence assessments for "general health claims," state that "general health claims must not be false or misleading and their accuracy must be established through an established methodology to meet the appropriate standard of evidence."[2] NHPs with "general health claims," however, can rely on supportive evidence where "there are challenges in determining the therapeutic effect."[2] These include "patterns of evidence" from multiple sources, "specific end-points," which focus on overall health/medicine system benefits, or "qualifications," which essentially relates to the hedging of health claims with grammar modality (e.g. "could," "likely," "may," etc.).[2] In sum, "general health claims," which include "general health maintenance claims," operate in an arguably ambiguous state of only suggestive or potential benefit, whether that benefit relates to a body's normal or ailment-suffering states.

The regulation of NHPs in Canada has faced scrutiny in both formal assessments and popular discourse.[3,4,13] Notably, the Office of the Auditor General's 2021 audit of Health Canada's NHP regulation program found that "Canada fell short of ensuring that products were safe and effective."[13] Issues were found in both approval and monitoring processes,

noting that “little” was done “to prevent poor information from being given to consumers about licensed natural health products.”[13] Additionally, analysis on a sample of licensed NHPs found 88% had been advertised with misleading label information, and 56% with misleading information related to health claims and consumer uses.[13] The report recommended improved monitoring and oversight mechanisms of NHP quality, labeling, and advertising and for strengthening compliance and enforcement tools. These recommendations were acknowledged and accepted by Health Canada.[3,13] In 2023, Health Canada extended the Protecting Canadians from Unsafe Drugs Act (Vanessa’s Law) to NHPs. This change increases Health Canada’s regulatory authority by granting it the ability to strengthen surveillance capacity, recall unsafe products, impose greater financial penalties, and mandate further product testing from manufacturers.[15] Furthermore, Health Canada is currently working towards improving NHP labelling requirements and instilling fees for pre-market evaluation, site licences, and rights to sell.[15,16]

References

1. Binns CW, Lee MK, Lee AH. Problems and Prospects: Public Health Regulation of Dietary Supplements. Annual Review of Public Health. 2018 Apr;39(1):403–20.

2. U.S. Food and Drug Administration. Questions and Answers on Dietary Supplements [Internet]. U.S. Food and Drug Administration; 2022 [cited 2024 Sep 5]. Available from: https://www.fda.gov/food/information-consumers-using-dietary-supplements/questions-and-answers-dietary-supplements

3. Health Canada. About Natural Health Product Regulation in Canada [Internet]. Canada.ca. Government of Canada; 2004 [cited 2024 Sep 5]. Available from: https://www.canada.ca/en/health-canada/services/drugs-health-products/natural-non-prescription/regulation.html

4. Jarry J. Office for Science and Society. 2023 [cited 2024 Sep 5]. The False Reassurance of Dietary Supplement Regulation. Available from: https://www.mcgill.ca/oss/article/critical-thinking-health-and-nutrition/false-reassurance-dietary-supplement-regulation

5. Office of Dietary Supplements. Dietary Supplement Health and Education Act of 1994 [Internet]. U.S. Department of Health & Human Services; 1994 [cited 2024 Oct 3]. Available from: https://ods.od.nih.gov/About/DSHEA_Wording.aspx

6. Federal Trade Commission. Health Products Compliance Guidance [Internet]. Federal Trade Commission. [cited 2024 Oct 3] Available from: https://www.ftc.gov/system/files/ftc_gov/pdf/Health-Products-Compliance-Guidance.pdf

7. U.S. Food and Drug Administration. Guidance for Industry: Substantiation for Dietary Supplement Claims Made Under Section 403(r) (6) of the Federal Food, Drug, and Cosmetic Act [Internet]. U.S. Food and Drug Administration Center for Food Safety and Applied

Nutrition; 2022. Available from: https://www.fda.gov/regulatory-information/search-fda-guidance-documents/guidance-industry-substantiation-dietary-supplement-claims-made-under-section-403r-6-federal-food

8. Department of Justice. Canada. Natural Health Products Regulations [Internet]. Minister of Justice; 2023 [cited 2024 Jun 14]. Available from: https://laws-lois.justice.gc.ca/PDF/SOR-2003-196.pdf

9. U.S. Food and Drug Administration. Structure/Function Claims [Internet]. U.S. Food and Drug Administration - Human Foods Program; 2024 [cited 2024 Sep 5]. Available from: https://www.fda.gov/food/nutrition-food-labeling-and-critical-foods/structurefunction-claims

10. U.S. Food and Drug Administration. FDA's Regulation of Dietary Supplements with Dr. Cara Welch [Internet]. FDA - Center for Drug Evaluation and Research; 2023 Dec 13 [cited 2024 Oct 2]; Available from: https://www.fda.gov/drugs/news-events-human-drugs/fdas-regulation-dietary-supplements-dr-cara-welch

11. U.S. Food and Drug Administration. FDA Launches New Dietary Supplement Education Initiative[Internet]. FDA - Center for Food Safety and Applied Nutrition; 2022 Jun 2 [cited 2024 Sep 5]; Available from: https://www.fda.gov/food/cfsan-constituent-updates/fda-launches-new-dietary-supplement-education-initiative

12. U.S. Food and Drug Administration. Consumer Alert on Regenerative Medicine Products Including Stem Cells and Exosomes [Internet]. FDA - Center for Biologics Evaluation and Research; 2020 Jul 22 [cited 2024 Sep 5]; Available from: https://www.fda.gov/vaccines-blood-biologics/consumers-biologics/consumer-alert-regenerative-medicine-products-including-stem-cells-and-exosomes

13. Health Canada. Report 2—Natural Health Products—Health Canada [Internet]. Government of Canada - Office of the Auditor General of Canada; 2021 [cited 2024 Sep 5]. Available from: https://www.oag-bvg.gc.ca/internet/English/parl_cesd_202104_02_e_43806.html

14. Health Canada. Pathway for Licensing Natural Health Products Making Modern Health Claims [Internet]. Health Canada; 2012 [cited 2024 Sep 5]. Available from: https://www.canada.ca/en/health-canada/services/drugs-health-products/natural-non-prescription/legislation-guidelines/guidance-documents/pathway-licensing-making-modern-health-claims.html

15. Health Canada. Protecting Canadians from Unsafe Drugs Act (Vanessa's Law) Amendments to the Food and Drugs Act (Bill C-17) [Internet]. www.canada.ca. Health Canada; 2013 [cited 2024 Sep 5]. Available from: https://www.canada.ca/en/health-canada/services/drugs-health-products/legislation-guidelines/protecting-canadians-unsafe-drugs-act-vanessa-law-amendments-food-drugs-act.html

16. Health Canada. Natural Health product regulation in Canada: Natural health product cost recovery Overview [Internet]. Health Canada;2023 [cited 2024 Sep 12]. Available from: https://www.canada.ca/en/health-canada/services/drugs-health-products/natural-non-prescription/regulation/cost-recovery.html

Supplementary Materials 2: Methods (detailed description)

On December 22, 2023, we searched "stem cells" on Amazon.com for all products, both sponsored and non-sponsored, listed on in the category "Health & Household" and sub-category "Vitamins, Minerals, and Supplements." This search returned 991 total product URLS, and all corresponding metadata: product name, company, number of ratings, rating score (average), price, format, and sponsored/not-sponsored designation. All non-functional URLs were removed as well as product duplicates, such as the same product appearing in different sizes (e.g. 3 pack, 6 pack, large bottle small bottle, etc.). Next, each unique product ad was verified to contain either an explicit mention of stem cells or stem cell adjacent language in relation to cells or cell activity (e.g. "regeneration," "rejuvenation," "renewal," "repair" etc.) Products that included neither explicit or adjacent stem cell language were excluded.

The finalized data set consisted of (N=184) stem cell supplements (170 (92.4%) non-sponsored) from 133 unique companies. Content analysis was performed on all remaining products, and applied to information appearing anywhere in an ad, including images. Prior to engaging the data, the content analysis approached was directed towards a focus on health claims, promotional product descriptors, and appeals to science/scientific evidence. As detailed in the introduction, there is a relationship between health claims and the objective of and requirement for supporting claims with scientific evidence. The first stage of coding included determining whether the products were currently available, available in Canada, and whether they included a defining description of stem cells. Products not "currently available" were included in the analysis as none of these products were listed as discontinued by the manufacturer. There was no indication as to when the products became unavailable or if their availability status was final. Next, analysis captured which health ailments the products claimed to address, which beneficial descriptors were used to describe products, whether science rhetoric or scientific evidence was used to demonstrate quality/efficacy, and whether health care practitioners, including health scientists were mentioned or appeared in images. Only explicit mentions (either in text or images) were included as substantiating evidence for each category, thus reducing all subjective interpretation. For example, in the category of appeals to scientific evidence, only direct mentions of, for example, "science," "scientists," "trials," or "research" were coded as present. More subjective interpretations of science-related images (e.g. DNA helices; space imagery, futuristic graphics) were not included. Explicit mentions of healthcare practitioners (e.g. "doctors," "physicians," "nurses," etc.) were included as were images of individuals wearing, for example, lab coats and stethoscopes. Two coders coded the dataset over a two-week period and met periodically to ensure there was no coding ambiguity.

Following coding completion, a third coder checked all coding, observing 90% accuracy. Additional findings observed by the third coder were verified and incorporated.
